# Amplified parabrachial nucleus activity in a rat model of trigeminal neuropathic pain

**DOI:** 10.1016/j.ynpai.2018.02.002

**Published:** 2018-03-01

**Authors:** Olivia Uddin, Paige Studlack, Titilola Akintola, Charles Raver, Alberto Castro, Radi Masri, Asaf Keller

**Affiliations:** a Department of Anatomy and Neurobiology, University of Maryland School of Medicine, 20 Penn St, HSF-II S251, Baltimore, MD 21201, United States; b Program in Neuroscience, University of Maryland School of Medicine, 20 Penn St, HSF-II S251, Baltimore, MD 21201, United States; c Department of Advanced Oral Sciences and Therapeutics, University of Maryland School of Dentistry, 650 W. Baltimore St, Baltimore, MD 21201, United States

**Keywords:** Chronic pain, Affective pain, Facial grimace, Chronic constriction injury, After-discharges

## Abstract

The parabrachial (PB) complex mediates both ascending nociceptive signaling and descending pain modulatory information in the affective/emotional pain pathway. We hypothesized that PB hyperactivity influences chronic pain behavior after trigeminal nerve injury in rats. Following induction of neuropathic pain using the chronic constriction injury of the infraorbital nerve (CCI-ION) model, rats displayed spontaneous markers of pain and mechanical hyperalgesia extending beyond the receptive field of the injured nerve. PB neurons recorded from rats with CCI-ION displayed amplified activity, manifesting as significantly longer responses to sensory stimuli, compared to shams. These findings suggest that chronic neuropathic pain involves PB hyperactivity.

## Introduction

Chronic pain is a major health issue affecting over a million people in the US and causing an annual economic burden of approximately $600 billion (Committee on Advancing Pain Research, 2011). Of significant concern is chronic neuropathic pain, which arises due to lesions or dysfunction in peripheral or central pain pathways.

Neuropathic pain affecting the trigeminal system, in particular, is frequently associated with negative affective states, including a high incidence of depression, anxiety, and sleep disorders (Wu et al., 2015; Mousavi et al., 2016; Smith et al., 2013). Patients with trigeminal neuralgia and traumatic trigeminal neuropathic pain also suffer from hypersensitivity to light touch and temperature, often accompanied by a more persistent, dull pain in the region of the face innervated by the damaged nerve (Gregg et al., 1979; Dubner et al., 1987; Love and Coakham 2001; Elias et al., 2014).

The parabrachial complex (PB) is a collection of nuclei at the junction of the midbrain and pons that mediates, in addition to pain, a variety of functions important to satiety, taste, arousal, respiratory control, and fluid and salt balance (Davern, 2014; Martelli et al., 2013; Hajnal et al., 2009). PB neurons respond robustly to noxious cutaneous and to visceral stimuli (Bernard et al., 1994; Bester et al., 1995; Gauriau and Bernard, 2002), and is an anatomical target of nociceptive neurons from both the trigeminal and spinal dorsal horn (Gauriau and Bernard, 2002; Spike et al., 2003; Saito et al., 2017). Nociceptive information from the periphery is relayed from PB to brain regions implicated in pain and affect, including the central nucleus of the amygdala, thalamus, zona incerta, hypothalamus, bed nucleus of the stria terminalis, and insular cortex (Gauriau and Bernard, 2002; Bianchi et al., 1998; Krukoff et al., 1993; Nakao et al., 2012). PB also shares reciprocal connections with regions comprising the descending pain modulatory system, including the prefrontal cortex, periaqueductal gray, and rostral ventral medulla (Buchanan et al., 1994; Roeder et al., 2016; Chen et al., 2017). Thus, PB is situated at the nexus of ascending and descending pain processing pathways.

This anatomical substrate suggests that PB may be involved in the pathogenesis of chronic pain. Consistent with this hypothesis, Matsumoto and colleagues (1996) report that both spontaneous and evoked activity of PB neurons are increased in arthritic rats. There are also reports of enhanced gene activity and metabolism in PB of rats with chronic constriction injury (CCI) of the sciatic nerve (Jergova et al., 2008; Mao et al., 1993).

We have recently shown that CCI of the infraorbital nerve (CCI-ION) results in a transition from acute to persistent pain that is dependent on central mechanisms (Okubo et al., 2013; Castro et al., 2017). We also suggested that this chronic, trigeminal pain might differ from somatic pain (Vos et al., 1994; Akintola et al., 2017), consistent with previous studies (Schmidt et al., 2015; Meier et al., 2014). Therefore, we tested the hypothesis that hyperactivity of PB neurons contributes to the pathogenesis of trigeminal chronic pain.

## Methods

### Animals

All procedures were conducted according to Animal Welfare Act regulations and Public Health Service guidelines and approved by the University of Maryland School of Medicine Animal Care and Use Committee. We studied 27 male Sprague-Dawley rats (375–475 g at time of electrophysiological recordings, ordered from Envigo, Indianapolis, IN) in these experiments: 7 sham-operated and 20 CCI-ION.

Animals were randomly allocated to experimental or control groups, as described in Kim and Shin (2014). In all experiments, the investigators were blinded to animal condition until data analysis was completed. Thus, allocation concealment, blinded conduct of the experiment, and blinded assessment of the outcomes were performed.

### Induction of chronic orofacial pain

We used a rodent model of neuropathic pain, evoked by chronic constriction of the infraorbital nerve (CCI-ION) (Benoist et al., 1999; Vos et al., 1994; Okubo et al., 2013; Castro et al., 2017; Akintola et al., 2017). Animals were first induced with 2% isoflurane and then injected intraperitoneally with ketamine (100 mg/kg)/xylazine (10 mg/kg). Depth of anesthesia was confirmed by the absence of blink and hind paw pinch withdrawal reflexes, and monitored periodically throughout the surgery. The animal was placed in a supine position on a sterile surgical platform, and an 8–10 mm long intraoral incision was made along the roof of the mouth next to left cheek, beginning distal to the first molar. The infraorbital nerve was freed from surrounding connective tissue and clearly visualized before loosely tying it with silk thread (4–0), 1–2 mm from where the nerve emerges from the infraorbital foramen. The wound was cleaned and closed using Vetbond tissue glue (3M products, St. Paul, MN). Animals were monitored and allowed to recover on a warm heating pad and then monitored daily as they recovered for 5–7 days in their home cage.

### Behavioral assessment of pain and hyperalgesia

To assess tactile sensitivity, rats were held loosely without restraint on the experimenter’s arm while von Frey filaments (North Coast Medical, Gilroy, CA) of varying forces were applied to the buccal region. Each animal was tested bilaterally and a response was defined as an active withdrawal of the head from the probing filament. We used the up-down method to determine withdrawal thresholds, as described previously (Chaplan et al., 1994; Dixon 1965). This approach requires ten to twelve stimuli to be applied. We also assessed tactile responses on the plantar surface of the hind paws in an up-and-down manner to calculate hind paw mechanical withdrawal thresholds. In addition, hind paw withdrawal latencies from an infrared beam in a Hargreaves apparatus (IITC, Woodland Hills, CA) were measured over five trials and then averaged to assess thermal hypersensitivity. The order of stimuli to ipsilateral and contralateral sides was randomized. We compared grouped data with Mann-Whitney *U* ranked-sum tests.

To assess ongoing pain we analyzed facial grimace behavior (Langford et al., 2010; Sotocinal et al., 2011; Akintola et al., 2017). Rats were placed in a square Plexiglas chamber (8″ × 8″ inches) with two opaque sides and two transparent sides. The chamber contained home-cage bedding, and video recordings were taken for 20 min from both transparent sides. The scoring of the facial expressions was done via a semi-automated procedure using the “Face Finder” application (Sotocinal et al., 2011), generously donated by J. Mogil. Face images were screened, labelled, scrambled and scored with the experimenter blinded to the treatment group and identity of each image. The grimace scale quantifies changes in a four (4) “action units”: orbital tightening, nose-cheek bulge, whisker tightening and ear position. Ten screenshots were selected for each animal, and on each image, each action unit was given a score of 0, 1, or 2, as previously described (Langford et al., 2010; Sotocinal et al., 2011; Akintola et al., 2017). Mean grimace scale scores were calculated as the average score across all the action units.

We excluded four rats that did not develop signs of pain: Their mechanical withdrawal thresholds and their RGS scores were indistinguishable from sham-injured rats. Some of the data described here consisting of mechanical withdrawal thresholds from facial stimulation and RGS scores for only the CCI-ION group were published, in part, in Akintola et al. (2017).

### Surgical preparation for electrophysiology

To maintain a constant level of light anesthesia (Level III-2, as defined by Friedberg et al., 1999), we implanted an intravenous catheter into the right jugular vein to deliver urethane (10% w/v solution in normal saline) throughout the electrophysiological experiments (Sceniak and Maciver, 2006). Following jugular catheterization, rats were placed in a stereotaxic frame with body heat maintenance, and a small craniotomy was made over the recording site to target PB (AP −9.2 and ML +1.9, relative to bregma, and DV −6.0 mm, relative to dural surface).

We did not include recordings from three CCI-ION rats whose anesthetic level was deeper than Level III-2 (Friedberg et al., 1999).

### In vivo electrophysiology

Using platinum-iridium recording electrodes (2–4 MΩ) produced in our laboratory, we recorded from the PB ipsilateral to injury. We isolated units responsive to noxious cutaneous stimuli, to dermatomes in both the head and body, and digitized the waveforms using a Plexon system (Plexon Inc., Dallas, TX). Search stimuli consisted of application of a wooden probe to these dermatomes. Upon encountering a cell responsive to noxious cutaneous stimulation we allowed the neuron to resume baseline firing rate before recording spontaneous activity for three minutes, after which we recorded neuronal response to noxious stimuli. We applied mechanical or thermal stimuli within the V2 dermatome, or mechanical stimuli to the plantar surface of the hind-paw. Mechanical stimulation was produced with a calibrated electronic aesthesiometer (IITC, Woodland Hills, CA) and thermal stimulation with a Picasso Lite dental surgical laser (AMD Lasers, Indianapolis, IN), set to alternate on/off in 30 ms cycles at 2 W for three seconds. We calibrated the laser output by implanting a micro-temperature probe in the whisker pad; laser pulses resulted in a heat stimulus of 56 ± 4°C. Five repetitions each of mechanical and thermal stimuli were applied, alternating between ipsilateral and contralateral stimuli. Subsequent stimuli were applied when the neuron recorded resumed firing at its baseline rate, with at least 8 s between each application. If cells exhibited after-discharges, the inter-stimuli interval was longer, to capture the entire after-discharge duration.

### Electrophysiology data analysis

Cells were sorted using Offiine Sorter (Plexon Inc., Dallas, TX) using dual thresholds and principal component analysis. We subsequently generated autocorrelograms in NeuroExplorer (Plexon Inc.) to confirm that each recording was of a single unit.

Responses to tactile stimuli were analyzed using custom Matlab (MathWorks, Natick, MA) routines. The routines were used to calculate the integral of the force applied by the electronic aesthesiometer, the firing rate during stimulus application, and the spontaneous firing rate. Evoked responses were computed and expressed as evoked firing rate normalized to spontaneous firing rate, divided by the stimulus force integral.

Responses to thermal stimuli were analyzed with custom Matlab routines, and significant responses were defined as firing activity exceeding the 99% confidence interval of the baseline firing rate. Peristimulus time histograms (PSTHs) were generated to analyze responses to successive stimuli.

We defined after-discharges—periods of sustained activity that outlast a stimulus presentation (Okubo et al., 2013)—as PSTH bins in which activity exceeded the 99% confidence interval for a period lasting at least 500 ms after stimulus offset.

A small proportion of PB neurons exhibited suppressed firing in response to a stimulus, followed by rebound spikes (described below). We defined periods of significant response suppression for each cell using a paired *t*-test to compare firing rates during a 5 s period before the stimulus with firing rates during the stimulus. Periods of rebound spiking were defined as PSTH bins in which activity during the period immediately following removal of the stimulus exceeded the 99% confidence interval.

### Histology

To identify recording sites, electrolytic lesions were made at the end of a recording session. We sectioned the fixed brain tissue into 80 μm-thick coronal sections that were stained with cresyl violet.

### Statistical analysis

We analyzed group data using GraphPad Prism version 7.00 for Mac (GraphPad Software, La Jolla CA). Data are presented, unless otherwise noted, as median values ± 95% confidence intervals (95% CI).

## Results

### Behavioral confirmation of pain and hyperalgesia after CCI-ION

#### Mechanical withdrawal thresholds lowered by CCI-ION in face and hind-paw

**Fig. 1:**
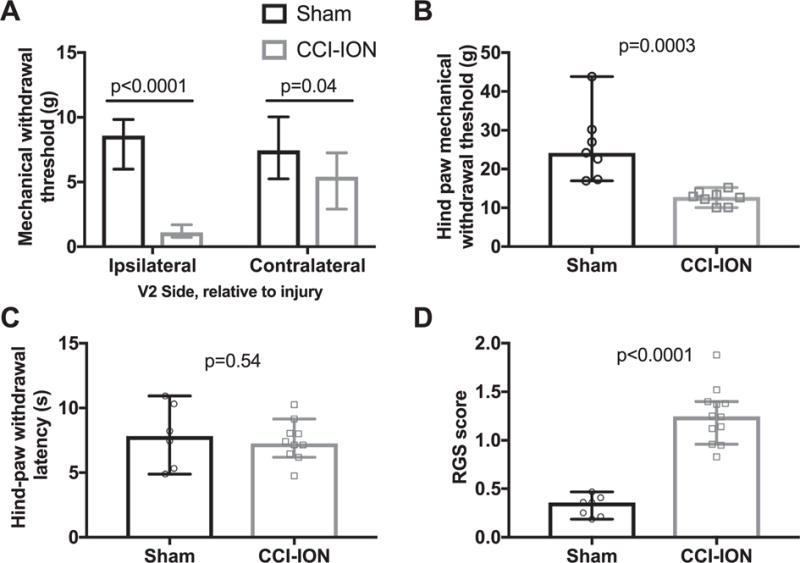
Behavioral confirmation of pain and hyperalgesia after CCI-ION. A: Mechanical withdrawal thresholds to facial stimuli are significantly reduced in CCI-ION rats (n = 14) compared to shams (ipsilateral Mann-Whitney *U* = 0; contralateral MW *U* = 24). B: Hind-paw withdrawal thresholds are significantly lower in CCI-ION rats compared to shams (*U* = 0). C: In contrast to mechanical thresholds, the latency to withdraw hind-paws from thermal stimuli is comparable in both groups (*U* = 24). D: Rat Grimace Scale scores are significantly elevated in CCI-ION rats compared to sham rats (*U* = 0).

Rats with CCI-ION had decreased thresholds for mechanical withdrawal from stimuli to the ipsilateral whisker pad. Median mechanical withdrawal threshold for rats (n = 7) after sham surgery was 8.58 g (95% CI = 5.99–9.83 g). In comparison, median mechanical withdrawal threshold of CCI-ION rats (n = 15) was 1.12 g (95% CI = 0.73–1.71 g; Mann-Whitney *U* = 0; ipsilateral sham vs. ipsilateral CCI-ION: p = 0.0001, Fig. 1A). The effect size was large, at 3.88 (Cohen’s d).

Mechanical withdrawal thresholds *contralateral* to the injured nerve, in the V2 region of CCI-ION rats were also lower than those of shams (CCI-ION: median = 5.4 g, 95% CI = 2.91–7.25; sham: median = 7.45 g, 5.25–10.03 g; Mann-Whitney *U* = 24; p = 0.04; Fig. 1A). The effect size was, again, large, at 1.04.

We measured hind paw mechanical withdrawal thresholds after injury to assess distal, secondary hyperalgesia following CCI-ION. The median withdrawal threshold of sham-operated rats (n = 7) was 24.2 g (95% CI = 17–43.8 g). In contrast, rats with CCI-ION (n = 8) had a median hind paw withdrawal threshold nearly 50% lower (effect size = 1.92): 12.83 g (95% CI = 10.06–15.24 g; Mann-Whitney *U* = 0, p = 0.0003, Fig. 1B), suggesting that secondary hyperalgesia may extend beyond the face in this model of trigeminal neuropathic pain.

#### Thermal sensitivity of hind paws was unaffected by CCI-ION

Unlike the mechanical withdrawal threshold results, thermal hind paw withdrawal thresholds measured with a Hargreaves apparatus were unaffected, with shams withdrawing at a median of 7.84 s (95% CI = 4.89–10.93 s) and CCI-ION rats withdrawing at 7.27 s (95% CI = 6.19–9.16 s; Mann-Whitney *U* = 24, p = 0.54, Fig. 1C). Thus, behavioral response to noxious thermal stimuli of the hind paws was not altered by trigeminal nerve injury.

#### Ongoing pain assessment with Rat Grimace Scores

To assess ongoing pain we used the Rat Grimace Scale (RGS) (Sotocinal et al., 2011). We have recently demonstrated that RGS is a reliable and sensitive metric for the assessment of ongoing pain in the CCI-ION model (Akintola et al., 2017). Nine days after injury, rats with CCI-ION demonstrated significantly higher RGS scores (CCI-ION: median = 1.25, 95% CI = 0.96–1.40, n = 12) than their sham-operated cohorts (median = 0.36, 95% CI 0.19–0.47, n = 7, Mann-Whitney *U* ranked-sum test, *U* = 0, p < 0.0001, Fig. 1D). The effect size was 3.91. This finding confirms that CCI-ION results in persistent, ongoing pain.

#### Increased after-discharges of PB neurons after CCI-ION

**Fig. 2:**
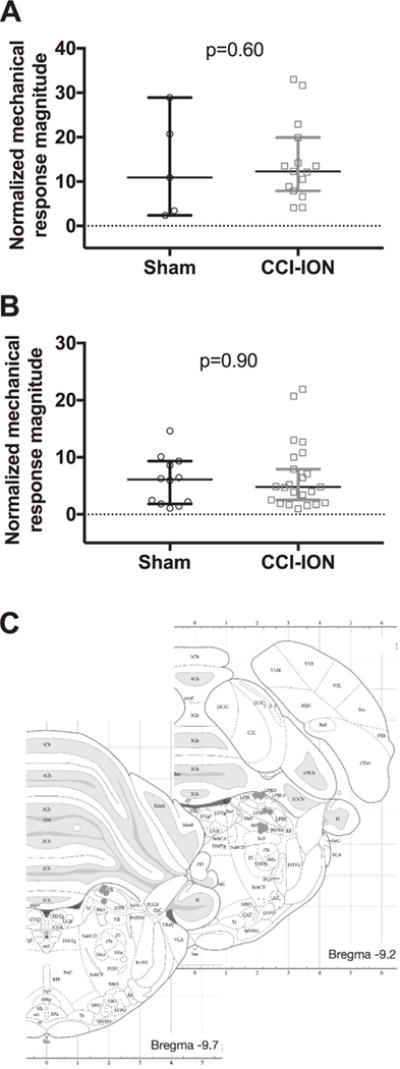
Parabrachial neurons respond to cutaneous stimuli. Responses to mechanical stimuli of the face (A; *U* = 31) and hind paws (B; *U* = 134) did not differ in sham and CCI-ION rats. C: Location of neurons recorded in PB marked by gray circle, each representing one to 5 neurons.

To assess electrophysiological correlates of the behavioral changes described above, we quantified the spontaneous activity of PB neurons, and their responses to noxious application of an electronic aesthesiometer to the face and hind paws. We correlated the output, in voltage, of the electronic aesthesiometer to force applied, in grams, and stimulated at forces greater than their behavioral withdrawal thresholds. The magnitude of responses to tactile stimuli (normalized to baseline firing rates, see Methods) did not differ for either facial or hind paw stimulation between injury condition groups. Cells from rats with sham injuries responded to facial stimuli with a median magnitude of 10.9 units (95% CI = 2.37–28.9, n = 5) while those from CCI-ION rats responded at a median magnitude of 12.3 units (95% CI = 7.89–19.9, n = 15; Mann-Whitney *U* = 31, p = 0.60; Fig. 2A). Similarly, cells in both conditions responded comparatively to hind-paw stimulation: shams (n = 12) had a median response magnitude of 6.11 units (95% CI = 1.83–9.34) and CCI-ION cells (n = 23) had a median response of 4.8 units (95% CI = 2.55–7.92; Mann-Whitney *U* = 134; p = 0.90; Fig. 2B).

**Fig. 3:**
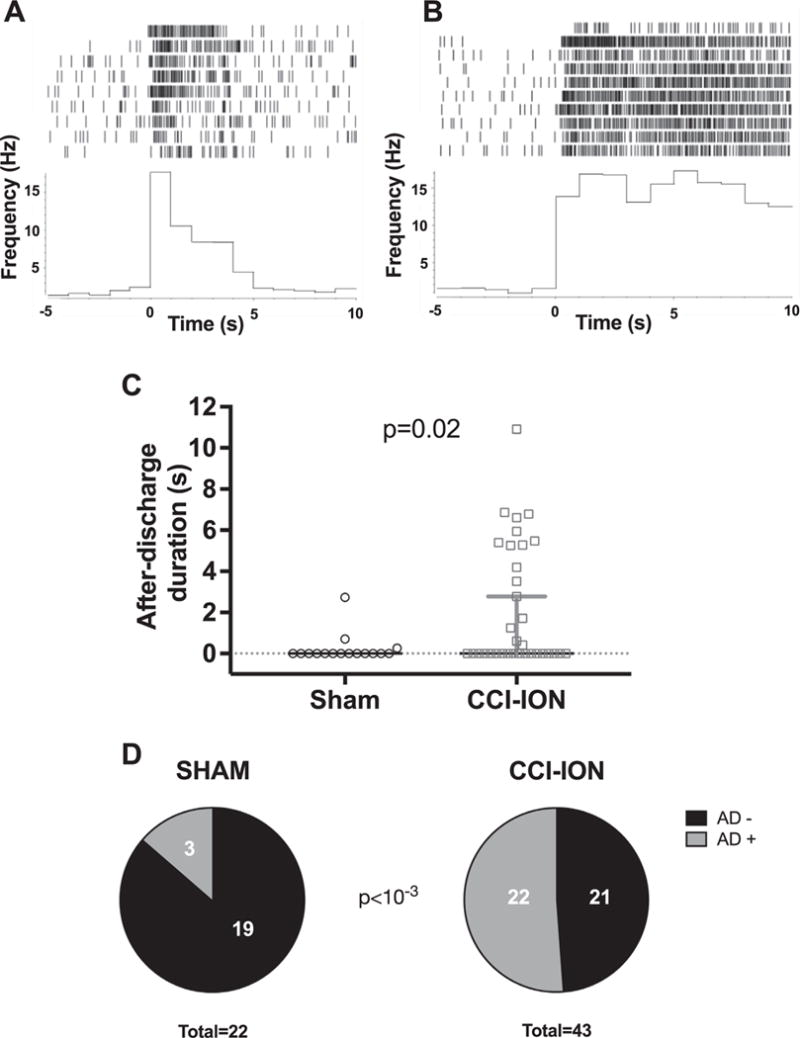
After-discharges in PB neurons are more prevalent and last longer after nerve injury. Representative post-stimulus time histogram (PSTH) and associated raster plot demonstrating firing in response to tactile stimulation (applied from time 0 to 3 s) to the hind-paw in a neuron from a sham animal (A) and from an animal with CCI-ION (B). C: After-discharge duration in PB neurons from CCI-ION rats (n = 43, median = 0.146 s, 95% CI = 1.12–2.893) are significantly longer that those from sham-operated rats (n = 22, median = 0 s, 95% CI = −0.1476–0.6415; Mann-Whitney *U* = 204; p = 0.02; Cohen’s d = 0.84). D: The proportion of neurons responding with after-discharges (AD) in CCI-ION rats was significantly higher than that in sham animals (two-tailed binomial test, p < 0.0001).

We previously reported that after-discharges—prolonged firing exceeding the duration of a stimulus—in trigeminal nucleus neurons are causally related to chronic pain after CCI-ION (Okubo et al., 2013). We recorded after-discharges in PB in response to tactile stimulation of either the hind-paws or the face in a subset of tactile responsive cells. Fig. 3A and B depict two representative PSTHs and raster plots comparing sham responses (A) to CCI-ION responses (B). Tactile stimulation lasts from 0 to 3 s in each PSTH, yet the representative neuron from the CCI-ION rat (Fig. 3B) continues firing at a sustained, high rate – displaying after-discharges.

PB neurons recorded from CCI-ION rats were nearly three times as likely to exhibit after-discharges, with 51.2% of tactile responsive neurons from CCI-ION rats and 13.6% of neurons from sham animals displaying after-discharges. This difference was statistically significant (binomial test, p < 0.0001, Fig. 3D).

PB neurons from CCI-ION rats (n = 43, median = 0.146 s, 95% CI = 1.12–2.893) had significantly longer after-discharges, compared to neurons from sham-operated rats (n = 22, median = 0 s, 95% CI = −0.1476 to 0.6415; Mann-Whitney *U* = 204; p = 0.02; Cohen’s d = 0.84). This analysis includes all neurons responsive to tactile stimuli—whether or not they displayed after-discharges; after-discharges in sham animals (n = 3) are too rare to generate meaningful comparisons with those in CCI-ION animals.

#### Subset of PB neurons depress firing to stimuli followed by rebound bursting

**Fig. 4:**
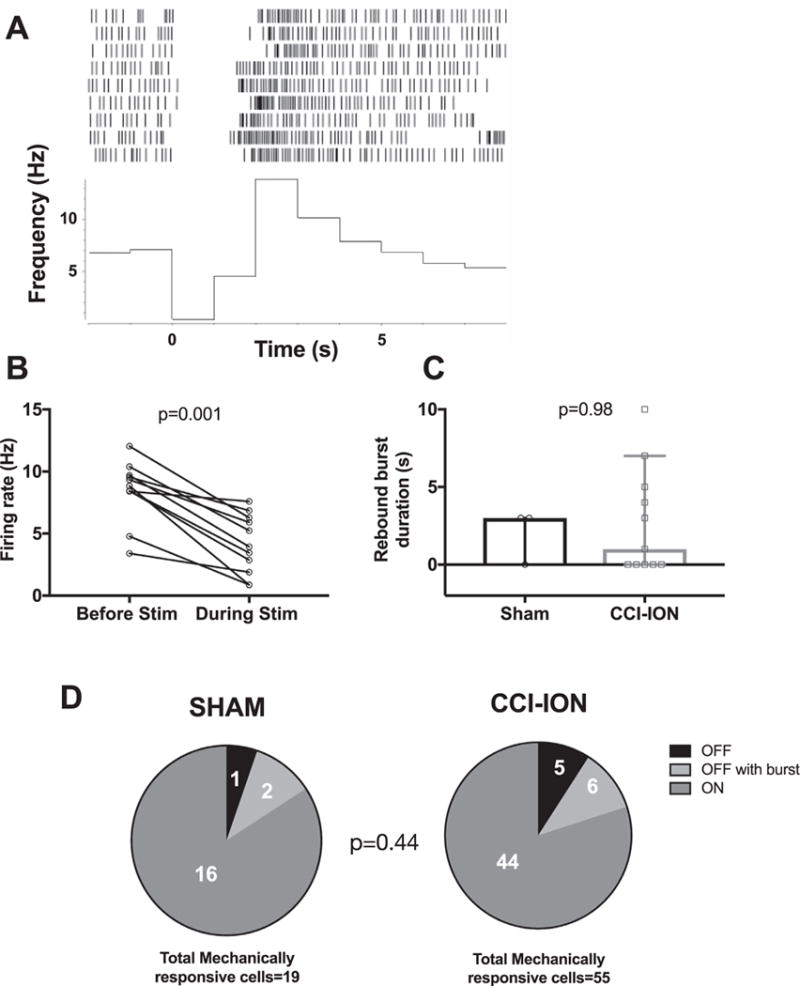
Depressed firing and rebound bursting cells in the PB. A: Example PSTH and raster plot demonstrating reduced firing during tactile stimulation (0–3 s), and rebound spiking. B: Suppressed firing in a subset of CCI-ION neurons (n = 11) during tactile stimulation; Wilcoxon test of paired samples. C: The duration of post-inhibitory rebound spikes did not differ between neurons from sham and CCI-animals (*U* = 15.5; medians and 95% CI). D: The proportion of OFF-cells with and without bursts, as a subset of all responsive neurons, was similar between sham and CCI-ION groups (Chi-square test; p = 0.44).

Another subset of the tactile responsive neurons showed a different response to mechanical stimulation: depressed responses with rebound bursting, as shown in the representative PSTH and raster plot in Fig. 4A. Three neurons from a single sham-operated animal displayed this response, whereas 11 neurons in PB of 6 CCI-ION rats suppressed firing (Fig. 4B). These proportions did not differ significantly between sham and CCI-ION groups (Chi square p = 0.44, Fig. 4D). The period of depressed firing quickly resolved after the removal of the mechanical stimulus, with some cells displaying a rebound burst (Fig. 4). The duration of the rebound burst was similar in sham and CCI-ION rats (sham: median = 3 s, 95% CI = 0–3 s; CCI: median = 1 s, 95% CI = 0–7 s; Mann-Whitney *U* = 15.5, p = 0.98; Fig. 4C).

#### Thermal response of PB cells are exaggerated after CCI-ION

**Fig. 5:**
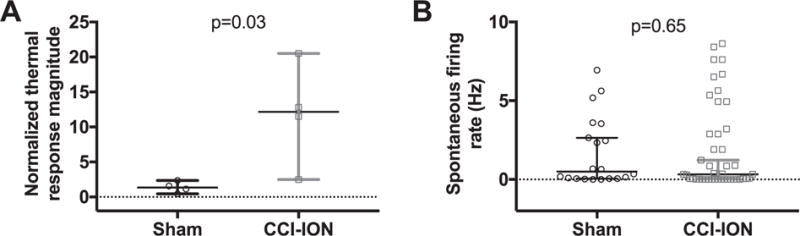
PB neurons in CCI-ION rats have amplified thermal responses. A: Response magnitude to thermal stimulation of the face was higher in neurons from CCI-ION animals, compared to shams (sham n = 4, CCI-ION n = 4, *U* = 0). B: Spontaneous firing rate was unchanged by CCI-ION (sham n = 18, CCI-ION n = 43, Mann-Whitney *U* = 358).

Only eight neurons responded to thermal stimulation of the face: 4 cells from sham-operated rats and 4 from CCI-ION rats. The response to thermal stimulation was significantly greater in the CCI-ION cells (median = 12.15, 95% CI = 2.49–20.52) than sham-operated cells (median = 1.35, 95% CI = 0.45–2.36; Mann-Whitney *U* = 0, p = 0.03; Fig. 5A; Cohen’s d = 1.99).

#### Spontaneous firing rate is similar in shams and CCI-ION PB neurons

Despite the hyperactivity of PB neurons after cutaneous stimulation in CCI-ION rats, spontaneous firing rates of neurons from sham-operated and CCI-ION rats did not differ. Neurons from sham animals (n = 18) had a median firing rate of 1.595 Hz (95% CI = 0.4649–2.724). Neurons in CCI-ION condition (n = 43) fired at a median firing rate of 1.912 Hz (95% CI = 1.041–2.783; *U* = 358, p = 0.65; Fig. 5B).

Approximately 11–25% of PB neurons in both sham-operated and CCI-ION groups, respectively, had no spontaneous firing, consistent with previous electrophysiological studies of PB neurons in uninjured animals (Bester et al., 1995; Bernard and Besson, 1990). When comparing only neurons that did fire spontaneously, there was still no significant difference in spontaneous firing rate between shams (n = 16, median = 0.50 Hz, 95% CI = 0.06–3.55) and CCI-ION PB neurons (n = 33, median = 0.86 Hz, 95% CI = 0.29–2.89; *U* = 246, p = 0.71).

Neurons recorded from both sham and CCI-ION rats were confirmed to be in the PB area (Fig. 2C), and were found in both the lateral and medial PB subnuclei.

## Discussion

### Development of pain behaviors in neuropathic trigeminal pain model

As in our previous studies, CCI-ION induced significant trigeminal hyperalgesia, as evident by the lowered thresholds for response to mechanical stimulation to the vibrissae pad (Akintola et al., 2017; Okubo et al., 2013; Castro et al., 2017). In addition, CCI-ION resulted in mechanical hypersensitivity in the hind paw (Fig. 1B), which has not been previously reported after CCI-ION. Expansion of hypersensitivity to areas beyond the receptive field of the injured nerve is consistent with the interpretation that maladaptive central changes occur in chronic pain (Woolf, 1983; Ziegler et al., 1999; Latremoliere and Woolf, 2009). The expansion of hyperalgesia to dermatomes distal from the trigeminal distribution may involve convergence of spinal and trigeminal inputs to the PB nucleus (Hayashi and Tabata 1990; Rodriguez et al., 2017).

Consistent with our recent report (Akintola et al., 2017), CCI-ION resulted also in significant increases in facial grimace scores, a reliable and sensitive metric for the assessment of ongoing pain (Langford et al., 2010; Sotocinal et al., 2011).

### Hyperexcitability of PB neurons

PB neurons responsive to noxious thermal stimuli fired more robustly after CCI-ION than after sham procedures. Cha et al. (2012) used the operant orofacial pain assessment device (OPAD) to assess thermal sensitivity of the face in rats subjected to CCI-ION, and found that thermal hyperalgesia persisted for at least eight weeks after injury, supporting our findings of hyperactive, thermally-responsive neurons in PB.

Contrary to our prediction, spontaneous firing rates and magnitudes of PB neuronal responses to noxious mechanical stimuli were not elevated in CCI-ION animals. Matusmoto and colleagues (1996) reported increased spontaneous firing rates of PB neurons in an arthritis model affecting mainly hind limb joints. It is possible that our results differ because of the different pain models studied. Matsumoto et al. studied a systemic, spinally-mediated chronic inflammatory pain state, whereas we induced chronic neuropathic pain with a trigeminal nerve injury. It is also possible that our studies focused on different populations of PB neurons: We included a large population of nociceptive neurons that had no spontaneous firing, whereas Matsumoto et al. (1996) report no spontaneously ‘silent’ neurons. Our findings are consistent with those of Bernard and Besson (1990) and Bester et al. (1995) who also reported PB neurons with very low spontaneous activity.

The most robust electrophysiological consequence of CCI-ION was the significant increase in after-discharges of PB neurons. CCI-ION was associated with robust hyperexcitability of PB neurons, evidenced as a 7-fold increased incidence of neurons exhibiting after-discharges, and a significant increase in the duration of these discharges (Fig. 3). One consequence of prolonged after-discharges may be temporal summation of synaptic inputs to neurons targeted by PB efferents, resulting in amplification of postsynaptic responses in these downstream neuronal targets. Therefore, because the PB nucleus projects to a number of nuclei associated with pain perception and with pain affects (see Introduction), the amplified after-discharges may be causally related to the hypersensitivity to tactile stimuli reported here after CCI-ION.

Indeed, we have previously demonstrated a causal relationship between after-discharges and pain metrics after CCI-ION (Okubo et al., 2013). We have yet to determine whether the after-discharges in PB reflect after-discharges in their presynaptic, SpVc inputs, or whether they reflect also changes intrinsic to PB neurons, or other pathophysiological mechanisms.

While aspects of PB perturbations in the CCI-ION model mirror changes seen in SpVc after the same injury (Castro et al., 2017; Okubo et al., 2013), there are several distinctions. Unlike PB neurons (present findings), SpVc neurons in animals with CCI-ION exhibit amplified spontaneous and evoked responses (Okubo et al., 2013). Whereas neurons in both regions exhibit after-discharges following CCI-ION, those in SpVc may last for minutes (Okubo et al., 2013), whereas those in PB last seconds (present findings). These differences suggest that PB may be subject to modulation from other brain regions after CCI-ION, such that its amplified responses do not entirely reflect the changes in its SpVc inputs.

A subset of PB cells responsive to mechanical stimulation exhibited stimulus-evoked depression followed by rebound bursts (Fig. 4). Approximately 15% of PB neurons recorded by Bernard and Besson (1988, 1990) displayed an inhibition of firing during cutaneous stimulation, but the authors made no mention of rebound bursting. Their recordings were made exclusively in uninjured animals, suggesting that these rebound spikes may reflect hyperexcitability related to the perception of pain (Bourinet et al., 1996; Choi et al., 2016; Rivera-Arconada and Lopez-Garcia, 2015). We found similar proportions of cells that depressed firing during mechanical stimulation in both sham and CCI-ION conditions (Fig. 4), but rebound bursting occurred more often and with longer duration in neurons from CCI-ION rats, suggesting that they may be hyper-excitable.

The pronounced hyperexcitability of PB neurons in this model of chronic pain—expressed as amplified after-discharges and rebound firing—suggest that CNS structures downstream from PB also reflect this amplified activity. The divergence of PB efferents to regions associated with the perception of affective pain, and to structures associated with descending pain modulation (see Introduction), strongly indicate that the PB nucleus is a key node in pain processing and in the pathogenesis of chronic pain.

## Abbreviations

PB: parabrachial complex
CCI-ION: chronic constriction injury of the infraorbital nerve
RGS: Rat Grimace Scale
PSTH: peristimulus time histograms
CI: confidence interval
SpVc: spinal trigeminal nucleus caudalis
